# Cytosolic/Plastid Glyceraldehyde-3-Phosphate Dehydrogenase Is a Negative Regulator of Strawberry Fruit Ripening †

**DOI:** 10.3390/genes11050580

**Published:** 2020-05-21

**Authors:** Ya Luo, Cong Ge, Min Yang, Yu Long, Mengyao Li, Yong Zhang, Qing Chen, Bo Sun, Yan Wang, Xiaorong Wang, Haoru Tang

**Affiliations:** College of Horticulture, Sichuan Agricultural University, Chengdu 611130, China; 13621@sicau.edu.cn (Y.L.); gecong945@sina.com (C.G.); yang137634@sina.com (M.Y.); 15982280577@sina.com (Y.L.); limy@sicau.edu.cn (M.L.); zhyong@sicau.edu.cn (Y.Z.); supnovel@sicau.edu.cn (Q.C.); 14099@sicau.edu.cn (B.S.); wangyanwxy@sicau.edu.cn (Y.W.); Wangxr@sicau.edu.cn (X.W.)

**Keywords:** glyceraldehyde-3-phosphate dehydrogenase, hydrogen peroxide, oxidative stress, strawberry, ripening

## Abstract

Cytosolic glyceraldehyde-3-phosphate dehydrogenase (GAPC) and plastid glyceraldehyde-3-phosphate dehydrogenase (GAPCp) are key enzymes in glycolysis. Besides their catalytic function, GAPC/GAPCp participates in the regulation of plant stress response and growth and development. However, the involvement of GAPC/GAPCp in the regulation of fruit ripening is unclear. In this study, *FaGAPC2* and *FaGAPCp1* in strawberries were isolated and analyzed. *FaGAPC2* and *FaGAPCp1* transcripts showed high transcript levels in the fruit. Transient overexpression of *FaGAPC2* and *FaGAPCp1* delayed fruit ripening, whereas RNA interference promoted fruit ripening and affected fruit anthocyanins and sucrose levels. Change in the expression patterns of *FaGAPC2* and *FaGAPCp1* also influenced the expression of several glycolysis-related and ripening-related genes such as *CEL1*, *CEL2*, *SS*, *ANS*, *MYB5*, *NCED1*, *ABI1*, *ALDO*, *PK*, and *G6PDH*, and H_2_O_2_ level and reduced glutathione (GSH)/glutathione disulfide (GSSG) redox potential. Meanwhile, metabolomics experiments showed that transient overexpression of *FaGAPCp1* resulted in a decrease in anthocyanins, flavonoids, organic acid, amino acids, and their derivatives. In addition, abscisic acid (ABA) and sucrose treatment induced the production of large amounts of H_2_O_2_ and inhibited the expression of *FaGAPC2*/*FaGAPCp1* in strawberry fruit. These results revealed that *FaGAPC2*/*FaGAPCp1* is a negative regulator of ABA and sucrose mediated fruit ripening which can be regulated by oxidative stress.

## 1. Introduction

Glyceraldehyde-3-phosphate dehydrogenase (GAPDH) is an important enzyme in the two sugar metabolism pathways of glycolysis and gluconeogenesis. The enzyme is highly conserved and recognized as a housekeeping gene present only in the cytosol [1]. However, in recent years, GAPDH has been found to have multiple localizations. Based on their different cellular localization, GAPDH can be divided into three isoforms: (1) cytosolic glyceraldehyde-3-phosphate dehydrogenase (GAPC)/plastid glyceraldehyde-3-phosphate dehydrogenase (GAPCp) which is located in the cytoplasm/plastid and specifically uses NAD^+^ as a coenzyme. It functions to phosphorylate glyceraldehyde-3-phosphate (G3P) and oxidizes it to form 1, 3-bisphosphoglycerate (1, 3-BPG). Strawberries (*Fragaria vesca*) have been shown to have four GAPCs including GAPC1, GAPC2, GAPCp1, and GAPCp2. The four consist of four identical subunits with high structural similarity [2]. (2) NP-GAPDH is located in the cytosol where it catalyzes the “bypass” reaction of glycolysis by directly oxidizing G3P to 3-phosphoglycerate and simultaneously producing NADPH [3]. (3) GAPA/GAPB is present in the chloroplast and requires NADP^+^ as a coenzyme and functions to reduce 1, 3-BPG to G3P. This enzyme is one of the important enzymes in the Calvin cycle and it is involved in the fixation of photosynthetic CO_2_ [4].

Recent studies have found that GAPC is a multifunctional protein, apart from the glycolytic function. The GAPC functional diversity is mainly due to its susceptibility to reversible redox posttranslational modifications (RPTM) on the catalytic cysteine [5] or protein–protein interactions altering its localization from the cytosol to the nucleus [6]. GAPC is very sensitive to H_2_O_2_ owing to the presence of a sensitive thiol switch and its abundance in the cytosol [7]. The concentration of GAPC in the cytosol is up to 240 μmol·L^−1^ and the potential thiol content is about 1 mmol·L^−1^ meaning that GAPC has a higher active thiol content than other redox proteins [8]. In a recent study, it was reported that E3 ubiquitin ligase (seven in absentia like 7, SINAL7) was involved in the relocation of the *AtGAPC1* [9]. In vitro, SINAL7 was shown to interact with AtGAPC1 whose nuclear localization signal drives the GAPC-SINAL7 complex into the nucleus to promote apoptosis. However, no AtGAPC1 was found in the nucleus of the SINAL7 deficient mutant. Furthermore, GAPC undergoes S-nitrosylation through the cellular NO donor, nitroso glutathione (GSNO), resulting in the formation of several redox-dependent modifications [10] which help GAPC to perform its function under oxidative stress.

Although the functional characterization of GAPC in the plant has been reported, little is known about GAPCp. In *Arabidopsis*, GAPCp has been demonstrated to be important in primary root growth and microspore development [11]. *GAPCp* double mutants (*gapcp1gapcp2*) also display a drastically reduced growth of the aerial part [12]. Moreover, GAPCp has been shown to participate in the production of ATP required for starch metabolism along with phosphoglycerol kinase in green and non-green plastids during dark periods [13]. However, whether GAPCp could be as susceptible as GAPC to oxidative stress in plants and its multi-functional properties is unknown.

The strawberry is an ideal model plant for the study of non-climacteric fruit. In recent years, a growing number of studies have focused on the regulation mechanism of ripening of strawberry fruit and this has mainly focused on the role of abscisic acid (ABA), auxin, cytokinin, ethylene (ETH), gibberellic acid (GA), and sucrose [14,15,16]. Except for hormones and sucrose, fruit development and ripening have been closely related to energy metabolism [17], respiratory metabolism [18], and the redox process [19]. Glycolysis is the central metabolic pathway in plants, and it has a direct effect on the above metabolism process. As one of the key enzymes in glycolysis, functional studies on *GAPDH* have also confirmed its involvement in plant growth, starch accumulation, energy metabolism, and seed germination. However, there are few reports about the relationship between glycolysis or *GAPDH* and fruit ripening, but there is also a report that the pentose phosphate pathway, which is closely related to glycolysis, is involved in the process of strawberry fruit ripening [20]. In our previous study, transcriptome data analysis showed that ABA and sucrose application accelerated strawberry fruit ripening and inhibited the expression of *GAPC2* and *GAPCp1* [15]. Therefore, there is a need for further research on how *GAPDH* affects fruit ripening in strawberries. Through a series of biochemical, transient gene expression, and metabonomic experiments, this study demonstrated that *GAPC2*/*GACPp1* is a negative regulator of fruit ripening in strawberries. This provides a promising gene candidate for manipulating fruit ripening in strawberries.

## 2. Materials and Methods

### 2.1. Plant Material

Strawberry (*Fragaria* × *ananassa* cv. Benihoppe) was grown in a plastic greenhouse under natural culture conditions in Chengdu, China. The experiment was performed in the spring of 2017. The fruits were harvested at different stages: Small green (SG, 7 days after anthesis), large green (LG, 14 d after anthesis), de-greening (DG, 18 d after anthesis), white (WT, 20 days after anthesis), initial red (IR, 23 days after anthesis), and full red (FR, 28 days after anthesis). In addition, the roots, shoot, leaves, flowers, and full red fruits of strawberry were collected. These materials were quickly frozen in liquid nitrogen and stored at −80 °C until use.

### 2.2. ABA and Sucrose Treatment

In total, about 500 secondary flowers from at least 100 plants were tagged and the fruits at the de-greening stage were sprayed with 95 μM ABA + 100 mM sucrose (ABA and sucrose were mixed in equal volumes) until dripping, and with water as the control. Eighteen berries were randomly sampled at 2-day intervals from the beginning of the treatment to the time at which all treated fruits reached the full-red stage for the determination of *FaGAPC2* and *FaGAPCp1* transcript levels.

### 2.3. Cloning of the FaGAPC2 and FaGAPCp1 Gene

Total RNA was extracted using a modified CTAB protocol [21]. Approximately 1 μg of total RNA was reverse transcribed to cDNA using a SMART™ RACE cDNA Synthesis Kit (TaKaRa, Nojihigashi, Japan). The primers used for *FaGAPC2* and *FaGAPCp1* gene cloning are listed in Table S1. The PCR reaction protocol was: 94 °C for 5 min, followed by 35 cycles at 94 °C for 30 s, 58 °C for 30 s and 72 °C for 2 min, with a final extension at 72 °C for additional 10 min.

### 2.4. Phylogenetic Analysis

The cloned *FaGAPC2* and *FaGAPCp1* genes sequences have been uploaded to GeneBank (BankIt2301415 BSeq#1 MN920478, BankIt2301449 BSeq#1 MN920479). The other protein sequences were downloaded from GDR and TAIR which included proteins from apples, pears, plums, peaches, and *Arabidopsis thaliana*. Among these proteins, the highly homologous sequences of GAPDH genes were identified using BioEdit with Local BLAST and e-value (1 × 10^−10^). A total of 31 GAPDH were obtained from SMART (http://smart.embl.de/) and NCBI CDD (https://www.ncbi.nlm.nih.gov/cdd/). The sequence alignment and the phylogenetic tree of GAPDH were constructed by using MEGA 7 with the neighbor-joining statistical method and bootstrap analysis (1000 replicates).

### 2.5. Plasmid Construction

The pTRV1, pTRV2, and pCAMeBIA1301 vectors were obtained from the horticultural plant biotechnology laboratory, College of Horticulture, Sichuan Agricultural University. The primers used for plasmid construction are listed in Table S2. For RNAi, the cDNA fragments of *FaGAPC2* (438 bp) and *FaGAPCp1* (532 bp) were inserted into the virus vector *BAMH* I-*Xba* I-cut pTRV2. For overexpression, the cDNA fragments of *FaGAPC2* (1048 bp) and *FaGAPCp1* (1318 bp) were inserted into the vector *BAMH* I-*Xba* I-cut pCAMBIA1301. These constructs were transformed into *Agrobacterium tumefaciens* strain GV3101 using the freeze–thaw method.

### 2.6. Transient Gene Expression in Strawberry Fruit

Transient gene expression assays in strawberry fruit were performed as described by Jia et al. [22]. A 5 mL culture of a single *Agrobacterium* colony was grown overnight at 28 °C in Luria–Bertani (LB) medium. The overnight cultures were inoculated into 50 mL of LB medium and grown at 28 °C overnight. The cells were harvested by centrifugation (5000× *g*, 5 min, 20 °C) and resuspended in an infiltration buffer (containing 10 mM MgCl_2_, 10 mM MES, 200 mM acetosyringone) to reach an OD_600_ of 1.0–2.0. Fruits at the DG (de-greening) stage were injected with the bacterial liquid using a 1 mL syringe. The fruit color was examined 5 days after the injection. Ten similar-sized fruits were used for the infiltration experiment. The overexpression experiment was carried out in December 2018 in winter and the silence experiment in April 2019 in spring.

### 2.7. Quantitative RT–PCR

A real-time fluorescence quantification PCR instrument (Bio-Rad, CFX Connect, Hercules, CA, USA) was used to perform real-time PCR amplifications. The primer sequences used for qPCR are shown in Table S3. The amplification program consisted of one cycle at 95 °C for 2 min, followed by 40 cycles of 94 °C for 20 s, 54 °C for 20 s, and 72 °C for 30 s. *FaActin* gene (AB116565) was used as the internal control. The qPCR experiment was repeated three times using biological replicates.

### 2.8. Determination of Total Anthocyanins, ABA, Sucrose, H_2_O_2_, Reduced Glutathione (GSH), Glutathione Disulfide (GSSG) Level, and GAPDH Enzyme Content

The total anthocyanin determination was conducted using the pH differential method [23]. Briefly, the mixed strawberry fruit (0.5 g) was extracted with 1.8 mL of cold 1% HCl-ethanol and centrifuged at 8000× *g* for 25 min at 4 °C, and then the supernatants were used for measuring the total anthocyanin content. Results were expressed as a milligram of pelargonidin 3-glucoside equivalents per 100 g of fresh weight.

Sucrose, GSH, and GSSG content were determined by the sucrose, GSH, and GSSG assay kit (Suzhou Keming Biotechnology Co., Ltd., Suzhou, China), the mixed strawberry fruit (0.2 g) was extracted with the respective extract provided in the kit, and centrifuged at 8000× *g* for 15 min, and then the supernatants were used for measuring the sucrose, GSH, and GSSG content in accordance with the manufacturer’s protocol, and the final reaction solution in absorbance was measured at 480, 412, and 412 nm, respectively.

The ABA content and GAPDH enzyme content were measured by the ABA and GAPDH enzyme content determination kit (Shanghai MLBIO Biotechnology Co., Ltd., Shanghai, China), respectively. The mixed strawberry fruit (0.2 g) was extracted with 1.8 mL PBS (phosphate buffer saline) for 30 min and centrifuged at 5000× *g* for 20 min, and then the supernatants were used for measuring the ABA content and GAPDH enzyme content in accordance with the manufacturer’s protocol by enzyme-linked immunosorbent assay.

H_2_O_2_ was measured as described by Cao et al. [24]. Briefly, mixed strawberry fruits (0.5 g) were extracted in 25 mL of 80% acetone for 1 h, followed by centrifugation at 12,000× *g* for 20 min. Finally, the concentration of H_2_O_2_ in the supernatants was measured. All samples were assayed as three independent biological replicates.

### 2.9. Treatment of H_2_O_2_ and Dithiothreitol (DTT)

The strawberry fruit discs at the DG stage were immersed in an equilibrium buffer (50 mM MES-Tris (pH = 5.5), 1 mM MgCl_2_, 1 mM EDTA, 5 mM CaCl_2_, 200 mM mannitol, 5 mM ascorbic acid) for 30 min [25], after which different volumes of H_2_O_2_ or DTT were added to the equilibrium buffer to make the concentration of H_2_O_2_ or DTT reach 1, 2, 4, and 8 mM. Next, the strawberry fruit discs were immersed in the above solutions for 4 h. Balanced buffers without H_2_O_2_ or DTT served as controls.

### 2.10. Metabolomic Analysis of FaGAPCp1 Overexpressing Fruits

The freeze-dried *FaGAPCp1* overexpressing fruits were crushed using a mixer mill (MM 400, Retsch) with a zirconia bead for 1.5 min at 30 Hz. 100 mg powder was weighted and extracted overnight at 4 °C with 1.0 mL 70% aqueous methanol. After centrifugation at 10,000× *g* for 10 min, the extracts were absorbed (CNWBOND Carbon-GCB SPE Cartridge, 250 mg, 3 mL; ANPEL, Shanghai, China) and filtered (SCAA-104, 0.22 μm pore size; ANPEL, Shanghai, China) and then subjected to LC-MS analysis.

### 2.11. HPLC Conditions

The sample extracts were analyzed using the LC-ESI-MS/MS system (HPLC, Shim-pack UFLC SHIMADZU CBM30A system, www.shimadzu.com.cn/; MS, Applied Biosystems 6500 Q TRAP). The analytical conditions were as follows: Column, Waters ACQUITY UPLC HSS T3 C18 (1.8 µm, 2.1 × 100 mm); solvent system, water (0.04% acetic acid): Acetonitrile (0.04% acetic acid); gradient program, 95:5 (*v/v*) at 0 min, 5:95 (*v/v*) at 11.0 min, 5:95 (*v/v*) at 12.0 min, 95:5 (*v/v*) at 12.1 min, 95:5 (*v/v*) at 15.0 min; flow rate, 0.40 mL·min^−1^; temperature, 40 °C; injection volume: 2 μL. The effluent was alternatively connected to the ESI-triple quadrupole-linear ion trap (Q TRAP)-MS.

### 2.12. ESI-Q TRAP-MS/MS

The LIT (Linear Ion Trap) and triple quadrupole (QQQ) scans were acquired on a triple quadrupole-linear ion trap mass spectrometer (Q TRAP), API 6500 Q TRAP LC/MS/MS System, equipped with an ESI Turbo Ion-Spray interface, operating in a positive ion mode and controlled by Analyst 1.6 software (AB Sciex). The operation parameters of the ESI source were as follows: Ion source, turbo spray; source temperature 500 °C; ion spray voltage (IS) 5500 V; ion source gas I (GSI), gas II (GSII), curtain gas (CUR) were set at 55, 60, and 25.0 psi, respectively; the collision gas (CAD) was high. Instrument adjustment and mass calibration were performed with 10 and 100 μmol/L polypropylene glycol solutions in QQQ and LIT modes, respectively. QQQ scans were acquired from multiple reaction monitoring (MRM) experiments with collision gas (nitrogen) set to 5 psi. For individual MRM transitions, DP and CE were further optimized. A specific set of MRM transitions were monitored for each period according to the metabolites eluted within this period.

### 2.13. Metabolite Profiling

Metabolite profiling was performed using a widely targeted metabolome method by Wuhan Metware Biotechnology Co., Ltd. (Wuhan, China). The freeze-dried samples were extracted as previously described and then analyzed using an LC–electrospray ionization (ESI)-MS/MS system. The extracts were absorbed with the CNWBOND Carbon-GCB SPE Cartridge, 250 mg, 3 mL (Shanghai ANPEL Scientific Instrument Co., Ltd., Shanghai, China). The metabolites were quantified using the multiple reaction monitoring (MRM) method.

### 2.14. Statistical Analyses

The data were analyzed by a one-way ANOVA test using SPSS software (Version 20; IBM, Almonk, NY, USA), and were expressed as mean ± SD. A *p* value of ≤ 0.05 was considered a statistically significant difference (Duncan’s multiple range test).

## 3. Results

### 3.1. Phylogenetic Tree and Distribution of GAPDH Family

**Figure 1:**
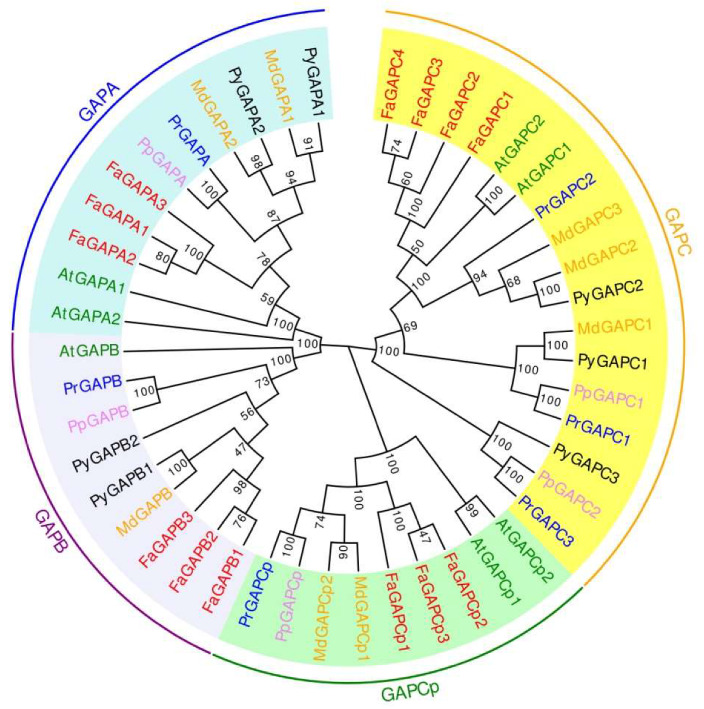
Phylogenetic analyses of glyceraldehyde-3-phosphate dehydrogenase (*GAPDH)* and its homologs. A neighbor-joining phylogenetic tree was constructed based on the amino acid sequence of GAPDH and its homologs from *Frugaria ananassa*, *Arabidopsis*, and several other species. Gene IDs were listed as: AtGAPA1 (AT3G04120), AtGAPA2 (AT1G12900), AtGAPB (AT1G42970), AtGAPC1 (AT3G04120), AtGAPC2 (AT1G13440), AtGAPCp1 (AT1G79530), AtGAPCp2 (AT1G16300), FaGAPA1 (augustus-masked-Fvb6-4-processed-gene-21.6-mRNA-1), FaGAPA2 (maker-Fvb6-1-snap-gene-6.77-mRNA-1), FaGAPA3 (maker-Fvb6-2-augustus-gene-273.24-mRNA-1), FaGAPB1 (maker-Fvb5-1-augustus-gene-175.48-mRNA-1), FaGAPB2 (maker-Fvb5-2-augustus-gene-165.20-mRNA-1), FaGAPB3 (maker-Fvb5-3-augustus-gene-117.23-mRNA-1), FaGAPC1 (maker-Fvb4-2-augustus-gene-68.54-mRNA-1), FaGAPC2 (maker-Fvb4-4-augustus-gene-66.43-mRNA-1), FaGAPC3 (maker-Fvb4-3-augustus-gene-99.43-mRNA-1), FaGAPC4 (maker-Fvb4-1-augustus-gene-135.60-mRNA-1), FaGAPCp1 (maker-Fvb3-2-augustus-gene-78.40-mRNA-1), FaGAPCp2 (maker-Fvb3-3-augustus-gene-68.46-mRNA-1), FaGAPCp3 (maker-Fvb3-4-augustus-gene-223.36-mRNA-1), PpGAPA (Prupe.3G315500), PpGAPB (Prupe.5G155800), PpGAPC1 (Prupe.5G155800), PpGAPC2 (Prupe.3G300600), PpGAPCp (Prupe.4G131700), MdGAPA1 (HF39109-RA), MdGAPA2 (HF32881-RA), MdGAPB (HF10057-RA), MdGAPC1 (HF08946-RA), MdGAPC2 (HF42750-RA), MdGAPC3 (HF35215-RA), MdGAPCp (HF11407-RA), MdGAPCp2 (HF22901-RA), PrGAPA (Prudul26A007753P1), PrGAPB (Prudul26A028000P1), PrGAPC1 (Prudul26A004526P1), PrGAPC2 (Prudul26A030279P1), PrGAPC3 (Prudul26A022505P1), PrGAPCp (Prudul26A013009P1), PyGAPA1 (pycom17g00030), PyGAPA2 (pycom111g00050), PyGAPB1 (pycom06g00570), PyGAPB2 (pycom16g26090), PyGAPC1 (pycom06g13820), PyGAPC2 (pycom13g09680), PyGAPC3 (pycom111g01370). The numbers indicate the bootstrap values calculated from 1000 replicate analyses.

GAPDH is divided into four categories: GAPA, GAPB, GAPC, and GAPCp. GAPA and GAPB are usually located in chloroplasts, while GAPC is located in cytoplasm, and GAPCp is located in non-green plastids. A phylogenetic tree of GAPDH protein from *Arabidopsis*, strawberries, apples, plums, and pears was constructed to show the phylogenetic relationship and classification of GAPDH genes. As shown in Figure 1, 13 strawberry GAPDHs, 8 apple GAPDHs, 7 pear GAPDHs, 6 plum GAPDHs, and 5 peach GAPDHs were identified. These proteins were classified into four cluster groups: GAPA (AtGAPA1, AtGAPA2, FaGAPA1, FaGAPA2, FaGAPA3, MdGAPA1, MdGAPA2, PyGAPA1, PyGAPA2, PrGAPA, PpGAPA), GAPB (AtGAPB, FaGAPB1, FaGAPB2, FaGAPB3, MdGAPB, PyGAPB1, PyGAPB2, PrGAPB, PpGAPB), GAPC (AtGAPC1, AtGAPC2, FaGAPC1, FaGAPC2, FaGAPC3, FaGAPC4, MdGAPC1, MdGAPC2, MdGAPC3, PyGAPC1, PyGAPC2, PrGAPC1, PrGAPC2, PrGAPC3, PrGAPC3, PpGAPC1, PpGAPC2), and GAPCp (AtGAPCp1, AtGAPCp2, FaGAPCp1, FaGAPCp2, FaGAPCp3, MdGAPCp1, MdGAPCp2, PrGAPCp, PpGAPCp).

### 3.2. ABA and Sucrose Induce H_2_O_2_ Production and Inhibit the Expression of FaGAPC2 and FaGAPCp1

**Figure 2:**
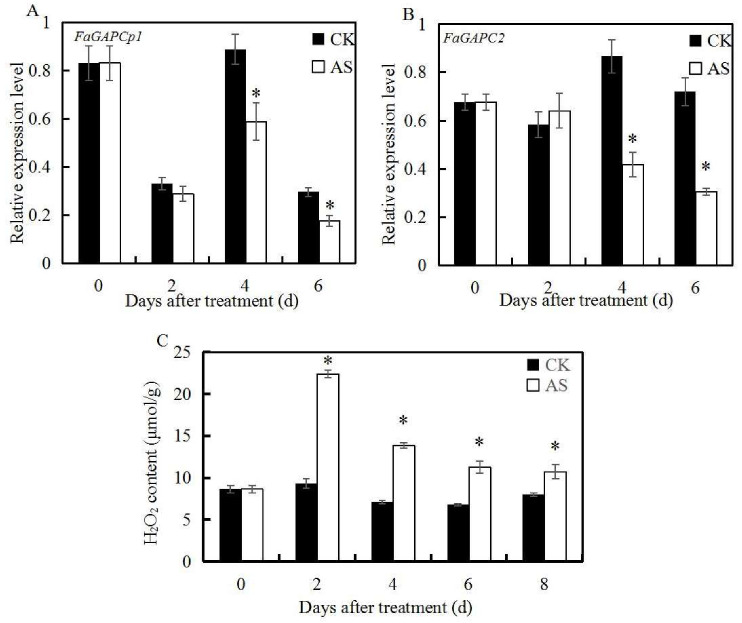
qRT-PCR analysis of *FaGAPC2* and *FaGAPCp1* genes and H_2_O_2_ content after abscisic acid (ABA) and sucrose treatment. (**A**) Changes in *FaGAPCp1* transcript levels in ABA + sucrose-treated fruit during the development and ripening of strawberry fruit; (**B**) changes in *FaGAPC2* transcript levels in ABA + sucrose-treated fruit during the development and ripening of strawberry fruit; (**C**) changes in H_2_O_2_ content in ABA + sucrose-treated fruit during the development and ripening of strawberry fruit. Strawberry was treated with 95 μM ABA + 100 mM sucrose (AS) and distilled water (CK) at the de-greening stage, respectively. Values are means ± SD of three biological replicates. Asterisks indicate statistically significant differences at *p* ≤ 0.05 as determined by Student’s *t*-test.

In our previous studies, transcriptome data analysis noted that mixed application of ABA and sucrose effectively promoted strawberry fruit ripening, and inhibited the expression of *GAPDH* [15]. The transcript levels of *FaGAPC2* and *FaGAPCp1* after the ABA and sucrose application were measured to further investigate the role of *FaGAPC2* and *FaGAPCp1* in fruit ripening in strawberries. The qRT-PCR results showed that the expression of *FaGAPC2* and *FaGAPCp1* in strawberry fruits were significantly down-regulated compared with the control, except for the second day after the treatment (Figure 2A,B). These findings suggested that *FaGAPC2* and *FaGAPCp1* are negative regulators in ABA and sucrose-mediated fruit ripening in strawberry fruits. In addition, H_2_O_2_ is an important signal molecule in plants. High H_2_O_2_ levels were detected in the ABA + sucrose-treated fruits (Figure 2C), which indicated that H_2_O_2_ plays a major role in fruit ripening in strawberries.

### 3.3. Spatial and Temporal Expression Profiles of Strawberry FaGAPC2 and FaGAPCp1

**Figure 3:**
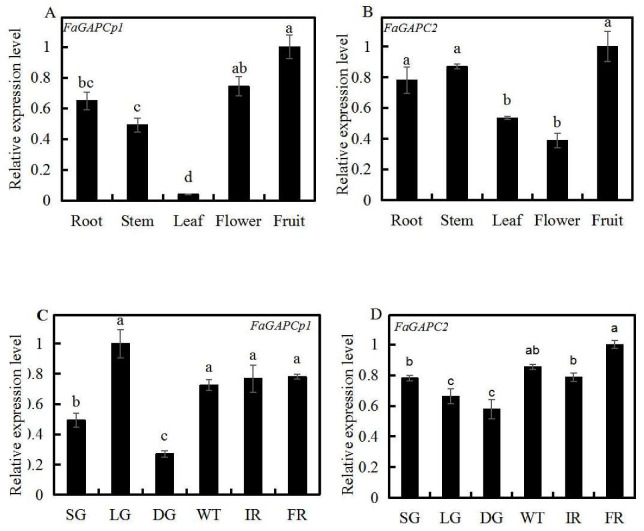
Transcript levels of *FaGAPC2* and *FaGAPCp1* in different tissues/organs and developmental stages. (**A**) *FaGAPCp1* transcript levels in root, stem, leaf, flower, and fruit; (**B**) *FaGAPC2* transcription levels in root, stem, leaf, flower, and fruit; (**C**) *FaGAPCp1* transcript levels in different developmental stages; (**D**) *FaGAPC2* transcript levels in different developmental stages. Small green (SG, 7 d after anthesis), large green (LG, 14 d after anthesis), de-greening (DG, 18 d after anthesis), white (WT, 20 d after anthesis), initial red (IR, 23 d after anthesis), and full red (FR, 28 d after anthesis). Values are means ± SD of three biological replicates. An overall significant difference (*p* ≤ 0.5) is represented by different lower-case letters as determined by Duncan’s multiple range test; a, b and ab show the significance of differences between groups of data.

qRT-PCR was used to determine the expression profiles of *FaGAPC2* and *FaGAPCp1* in different tissues/organs and at different stages of fruit development. The results showed that *FaGAPC2* and *FaGAPCp1* were expressed in all tested tissues/organs types (Figure 3). A high expression of *FaGACPp1* and *FaGAPC2* was detected in the fruit (Figure 3A,B). When accessed at different developmental stages, the transcript level of *FaGAPCp1* was lower at the SG (small green) and DG stages, followed by an increase, remaining at a higher transcript level from the WT (white) to FR (full red) stages (Figure 3C). The transcript level of *FaGAPC2* decreased from the SG to DG stages, followed by an increase, which was higher at the FR stage (Figure 3D).

### 3.4. Overexpression of FaGAPC2 and FaGAPCp1 Inhibits Strawberry Fruit Ripening

**Figure 4:**
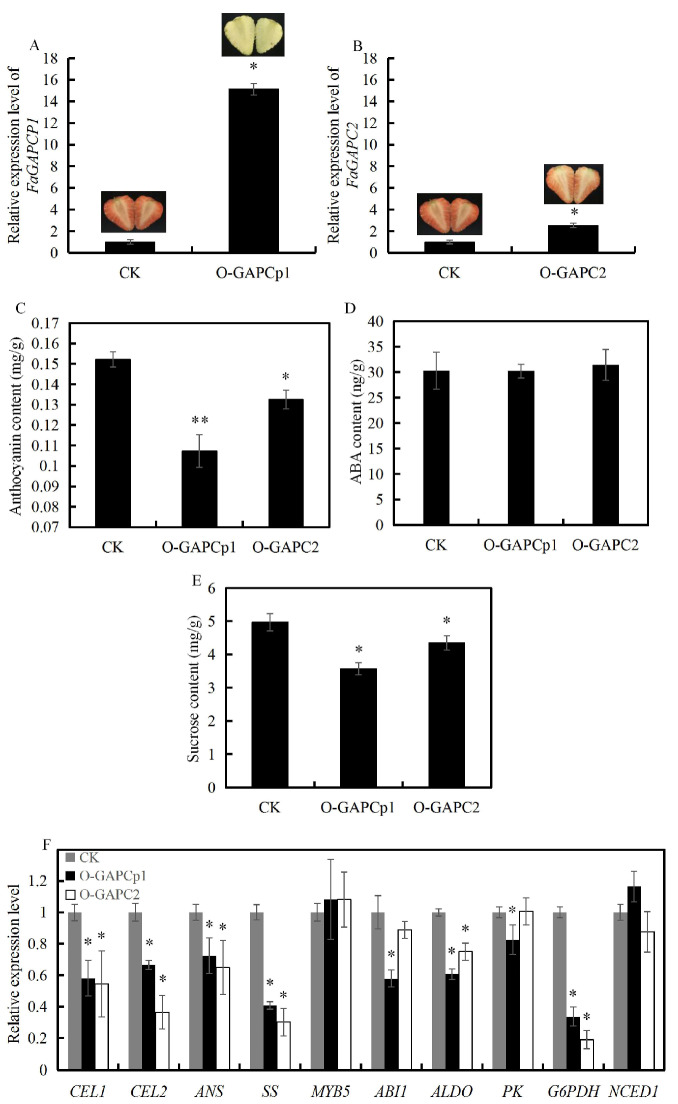
Overexpression for the *FaGAPC2* and *FaGAPCp1* genes in strawberry fruits. (**A**) Transcript levels of *FaGAPCp1* in the control, *FaGAPCp1* overexpression fruit by qRT-PCR; (**B**) transcript levels of *FaGAPC2* in the control, *FaGAPC2* overexpression fruit by qRT-PCR; (**C**) the total anthocyanins content in the control, *FaGAPC2* and *FaGAPCp1* overexpression fruit; (**D**) the ABA content in the control, *FaGAPC2* and *FaGAPCp1* overexpression fruit; (**E**) the sucrose content in the control, *FaGAPC2* and *FaGAPCp1* overexpression fruit; (**F**) transcript levels of ripening-related genes in the overexpression fruits and control. CK: control fruit; O-GAPCp1: *FaGAPCp1* overexpression fruit; O-GAPC2: *FaGAPC2* overexpression fruit. Data are means (±SD) obtained from three biological replicates. Asterisks indicate statistically significant differences at *p* ≤ 0.05 as determined by Student’s *t*-test.

To investigate the role of *FaGAPC2* and *FaGAPCp1* in strawberry fruit ripening, overexpression of *FaGAPC2* and *FaGAPCp1* via agro-infiltration provides a quick assay to test gene function. Accordingly, *35S:: FaGAPCp1* and *35S:: FaGAPC2* were shown to be infiltrated into the strawberry fruit. The qRT-PCR results showed that *FaGAPC2* and *FaGAPCp1* had higher expression in the fruit (Figure 4A,B). In addition, *35S:: FaGAPCp1* and *35S:: FaGAPC2* inhibited fruit coloring in strawberries (Figure 4A,B) with lower anthocyanins content (Figure 4C), and the coloring effect of *35S:: FaGAPCp1* was better than that of *FaGAPC2*. The results of ABA and sucrose content showed that ABA content did not change significantly (Figure 4D) and sucrose level decreased to about 0.87-fold and 0.72-fold (Figure 4E) in the *FaGAPC2* and *FaGAPCp1* overexpressed fruit compared with that of the control, respectively. Furthermore, qRT-PCR was used to measure the transcript levels of a set of glycolysis-related and ripening-related genes, including cell wall-related genes (cellulase 1, *CEL1* and cellulase 2, *CEL2*), which can decompose the cellulose in strawberry fruit and influence fruit ripening and softening [26], sucrose synthase (*SS*), anthocyanidin synthase (*ANS*), *MYB5*, which is an important transcription factor that affects strawberry fruit ripening by regulating anthocyanin synthesis [27], ABA biosynthesis gene (*NCED1*), ABA insensitive gene (*ABI1*), fructose-bisphosphate aldolase (*ALDO*), pyruvate kinase (*PK*), and glucose-6-phosphate dehydrogenase (*G6PDH*). The results (Figure 4F) showed that *CEL1*, *CEL2*, *SS*, *ANS*, *ALDO*, and *G6PDH* were all down-regulated, and *MYB5* and *NCED1* had no significant difference compared with that of the control fruit. In addition, the transcript levels of *PK* and *ABI1* decreased significantly only in the *FaGAPCp1* overexpressed fruit. These changes in sucrose and anthocyanins content and the expression of glycolysis, anthocyanins, cell wall-associated, and ABA-responsive genes in overexpressed strawberry fruit led to the inhibition of fruit ripening.

### 3.5. Transient Knock-Down of FaGAPC2 and FaGAPCp1 Promotes Strawberry Fruit Ripening

**Figure 5:**
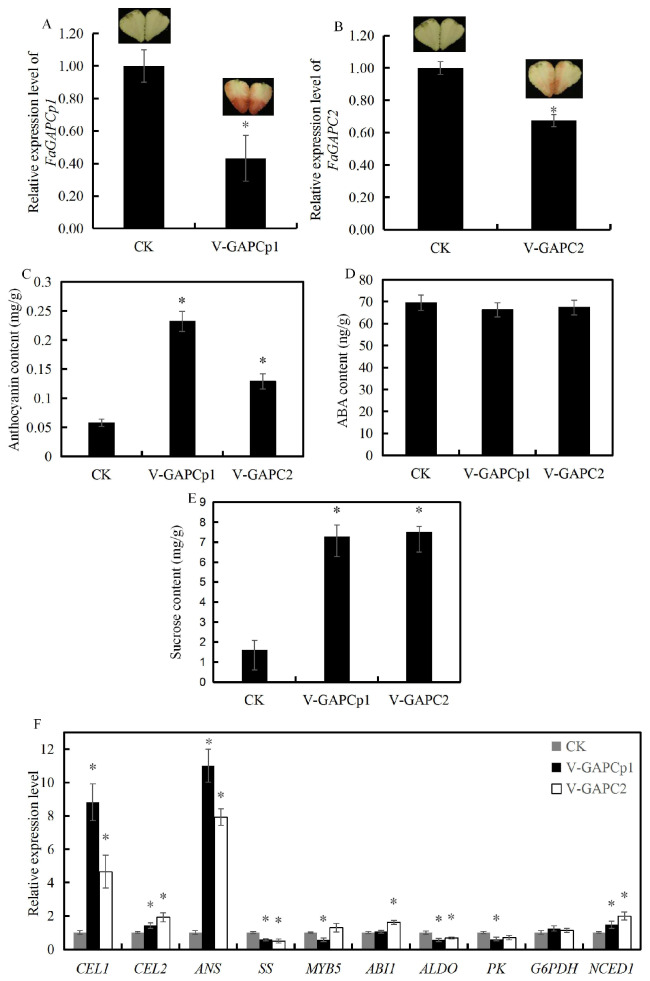
VIGS (virus induced gene silencing) for the *FaGAPC2* and *FaGAPCp1* genes in strawberry fruits. (**A**) Transcript levels of *FaGAPCp1* in the control, *FaGAPCp1*-RNAi fruit by qRT-PCR; (**B**) transcript levels of *FaGAPC2* in the control, *FaGAPC2*-RNAi fruit by qRT-PCR; (**C**) the total anthocyanins content in the control, *FaGAPC2*-RNAi, and *FaGAPCp1*-RNAi fruit; (**D**) the ABA content in the control, *FaGAPC2*-RNAi, and *FaGAPCp1*-RNAi fruit; (**E**) the sucrose content in the control, *FaGAPC2*-RNAi, and *FaGAPCp1*-RNAi fruit; (**F**) transcript levels of ripening-related genes in the RNAi fruits and control. CK: control fruit; V-GAPCp1: *FaGAPCp1*-RNAi fruit; V-GAPC2: *FaGAPC2*-RNAi fruit. Data are means (±SD) obtained from three biological replicates. Asterisks indicate statistically significant differences at *p* ≤ 0.05 as determined by Student’s *t*-test.

Transient RNAi was performed to knock-down *FaGAPC2* and *FaGAPCp1* to test whether *FaGAPC2* and *FaGAPCp1* were involved in the regulation of fruit ripening in strawberry. The study results revealed that it successfully resulted in down-regulation of *FaGAPC2* and *FaGAPCp1* (Figure 5A,B). Transient knock-down of *FaGAPC2* and *FaGAPCp1* dramatically promoted strawberry fruit coloring (Figure 5A,B) and anthocyanins accumulation (Figure 5C), and the coloring effect of *FaGAPCp1* was better than that of *FaGAPC2*. There was no significant change in the ABA content (Figure 5D) and sucrose content was much higher in *FaGAPC2-RNAi* and *FaGAPCp1-RNAi* fruit than in the control (Figure 5E). The qRT-PCR analysis showed that the transcript levels of glycolysis-related and ripening-related genes *CEL1*, *CEL2*, *ANS,* and *NCED1* were significantly up-regulated, whereas *SS* and *ALDO* were significantly down-regulated, and *G6PDH* had no significant difference compared with that of the control fruit (Figure 5F). However, a similar expression pattern of *AlDO* and *SS* in *FaGAPC2*/*FaGAPCp1* overexpression and silenced fruits was observed. These results indicated that the change of *FaGAPC2*/*FaGAPCp1* transcript levels might decrease the expression of *ALDO* and *SS*. In addition, *MYB5* and *PK* were down-regulated in the RNAi-*FaGAPCp1* fruit, while *ABI1* was up-regulated only in the RNAi-*FaGAPC2* fruit (Figure 5F). Therefore, these findings suggested that the *FaGAPC2* and *FaGAPCp1* genes influenced sucrose and anthocyanins content, and caused some anthocyanins, cell wall, and glycolysis-related genes to regulate fruit ripening.

### 3.6. Transient FaGAPC2 and FaGAPCp1 Expression and Oxidative Stress

**Figure 6:**
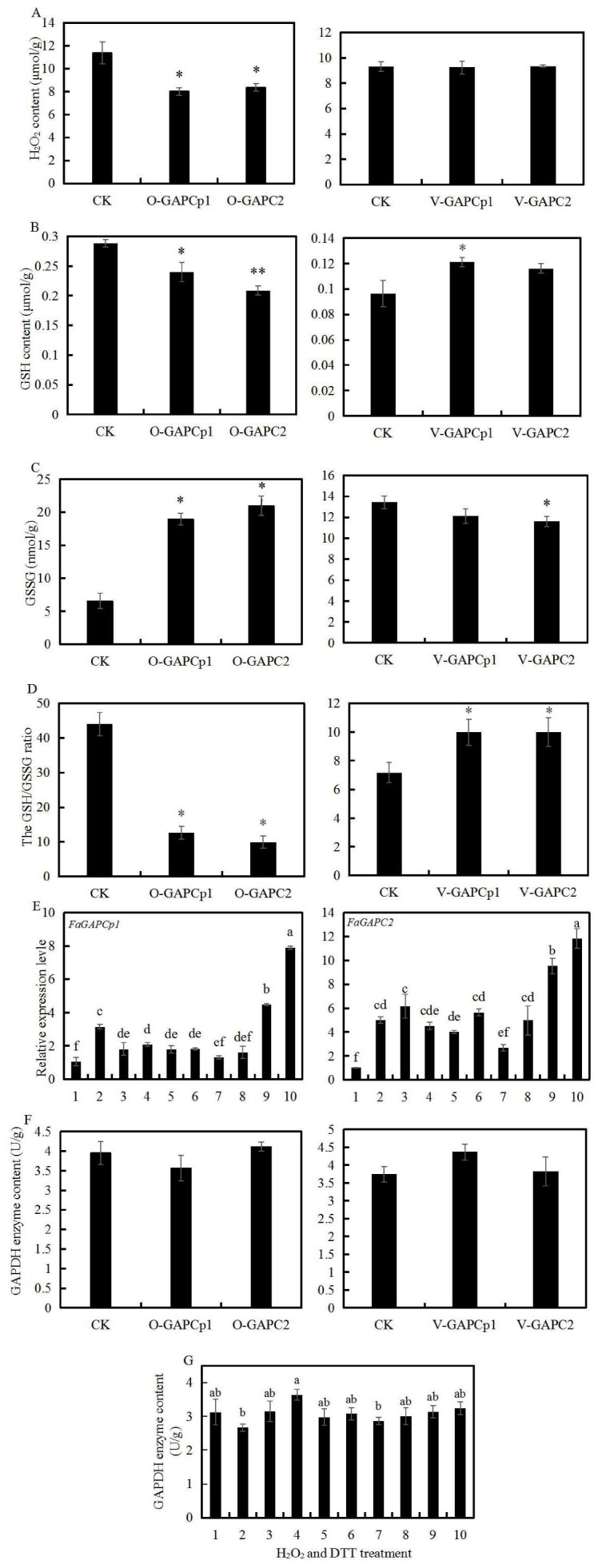
The interplay between *FaGAPC2* and *FaGAPCp1* functions and oxidative stress. (**A**) The H_2_O_2_ content in the control and transient *FaGAPC2* and *FaGAPCp1* expressing fruits; (**B**) the reduced glutathione (GSH) content in the control and transient *FaGAPC2* and *FaGAPCp1* expressing fruits; (**C**) the glutathione disulfide (GSSG) content in the control and transient *FaGAPC2* and *FaGAPCp1* expressing fruits; (**D**) the GSH/GSSG ratios in the control and transient *FaGAPC2* and *FaGAPCp1* expressing fruits; (**E**) transcript levels of *FaGAPCp1* and *FaGAPC2* at different concentrations of H_2_O_2_ and DTT treatment; (**F**) the GAPDH enzyme content in the control and transient *FaGAPC2* and *FaGAPCp1* expressing fruits; (**G**) the GAPDH enzyme content at different concentrations of H_2_O_2_ and DTT. Number 1 in abscissa, control 1, strawberry fruit discs at DG (de-greening) stage before treatment; 2, control 2, strawberry fruit discs were immersed in balanced buffers for 4 h; 3–6: Strawberry fruit discs treated with 1, 2, 4 and 8 mM H_2_O_2_ for 4 h, respectively; 7–10: Strawberry fruit discs treated with 1, 2, 4 and 8 mM DTT for 4 h, respectively. Data are means (±SD) obtained from three biological replicates. Asterisks indicate statistically significant differences at *p* ≤ 0.05 as determined by Student’s *t*-test; a, b and ab show the significance of differences between groups of data.

The production or enhanced availability of reactive oxygen species (ROS) have traditionally been grouped as oxidative stress. H_2_O_2_ is one of the important ROS in plants, and GAPDH is very sensitive to H_2_O_2_. The H_2_O_2_ content, GSH and GSSG, and GAPDH enzyme content in transient *FaGAPC2* and *FaGAPCp1* expression fruit were measured to investigate the relationship between GAPDH and oxidative stress. Compared to the respective controls, H_2_O_2_ content was decreased in *FaGAPC2* and *FaGAPCp1* overexpressed fruits, but no significant differences were observed between the RNAi fruit and the control fruit (Figure 6A). GSH is the main substance scavenging H_2_O_2_ in plants. GSH content was lower in *FaGAPC2* and *FaGAPCp1* overexpressed fruits and higher in RNAi-*FaGAPCp1* fruit, but no changes were observed in RNAi-*FaGAPC2* fruit (Figure 6B). GSSG is the oxidized form of GSH, the content of which was increased in both overexpressed fruits, while decreased in RNAi-*FaGAPC2* fruit, and showed no significant difference in RNAi-*FaGAPCp1* fruit compared with that of the control fruit (Figure 6C). The GSH/GSSG ratio showed that *FaGAPCp1* and *FaGAPC2* overexpression decreased the GSH/GSSG ratios of strawberry fruit compared with that of the control, but their silencing increased GSH/GSSG ratios (Figure 6D). These results indicated that transient *FaGAPC2* and *FaGAPCp1* expression altered H_2_O_2_ content and GSH/GSSG pool, and this was associated with fruit ripening in strawberry.

In addition, qRT-PCR was used to measure the transcript levels of *FaGAPC2* and *FaGAPCp1* after different concentrations of H_2_O_2_ and DTT treatment. The results in Figure 6E show that H_2_O_2_ treatment had significant inhibitory effects on *FaGAPCp1* expression, but not on *FaGAPC2*, and 4 DTT and 8 mM DTT treatment increased the expression of *FaGAPC2* and *FaGAPCp1*. This suggested that the expression of *FaGAPC2* and *FaGAPCp1* is regulated by oxidative stress. Neither transient *FaGAPC2* and *FaGAPCp1* expression (Figure 6F) nor oxidative stress (Figure 6G) significantly altered the GAPDH enzyme content except for the 4 mM H_2_O_2_ treatment.

### 3.7. Overexpression of FaGAPCp1 Inhibits Ripening-Related Metabolism

**Figure 7:**
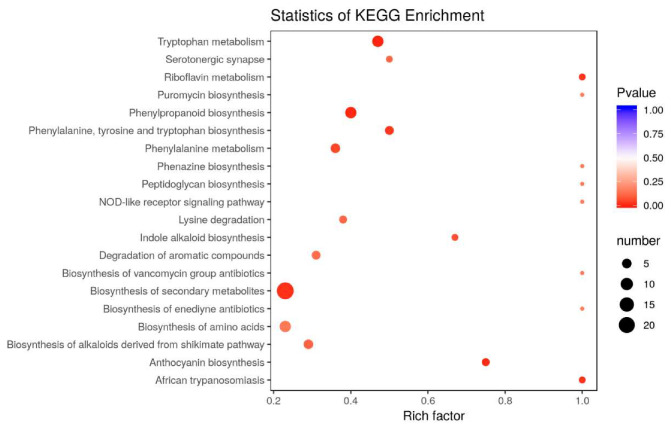
Top 20 enriched Kyoto Encyclopedia of Genes and Genomes (KEGGs) among the annotated differential metabolite. The *X*-axis represents the enrichment factor corresponding to each pathway, the *Y*-axis indicates the KEGG pathway. The color of the spot corresponds to different *p*-value ranges and the size of the spot represents the number of different metabolites enriched. A *p*-value less than 0.05 was significantly enriched.

Previous results have shown that *FaGAPC2* and *FaGAPCp1* are involved in fruit ripening in strawberries, and transient *FaGAPCp1* gene expression had a better effect than *FaGAPC2* on fruit ripening. Therefore, metabolomics was performed to analyze the transient *FaGAPCp1*-overexpression fruits and to test how GAPDH regulates strawberry fruit ripening at the metabolic level. The top 20 enriched Kyoto Encyclopedia of Genes and Genomes (KEGG) pathways among the annotated differential metabolites were selected (Figure 7). The three top-most enriched pathway terms were “biosynthesis of secondary metabolites”, “phenylpropanoid biosynthesis”, and “tryptophan metabolism”.

## 4. Discussion

### 4.1. FaGAPC2 and FaGAPCp1 as a Negative Regulator in the Regulation of Strawberry Fruit Ripening

Glycolysis is known to be an important metabolic pathway providing energy and precursors for fatty-acid and amino-acid synthesis [28]. GAPDH is also one of the key enzymes in glycolysis. Therefore, the change of *GAPDH* expression may affect the change of the whole metabolic level in plants. In recent years, this enzyme has been found to respond positively to various biotic and abiotic stresses [10,29,30,31] and is also involved in plant growth and development [32]. However, whether GAPDH plays a role in fruit ripening is unclear.

It is well known that ABA is the core signal in the regulation of strawberry fruit ripening, and sucrose acts as a signal to induce ABA accumulation and promote strawberry fruit ripening by ABA-dependent and ABA-independent pathways [33,34]. Therefore, the ripening process of strawberry fruit is as a result of the co-regulation of ABA and sucrose. In this study, ABA and sucrose treatment inhibited the transcript levels of *FaGAPC2* and *FaGAPCp1* (Figure 2A,B). Transient knock-down of *FaGAPC2* and *FaGAPCp1* promoted strawberry fruit ripening with increased anthocyanin and sucrose levels (Figure 5C,E), and overexpression of *FaGAPC2* and *FaGAPCp1* contributed to the opposite result (Figure 4C,E). This suggested that *FaGAPC2* and *FaGAPCp1* are negative regulators involved in the regulation of fruit ripening in strawberries. This was further supported by expression analysis of a set of ripening-related genes (Figure 4F and Figure 5F), including pigment and cell wall-related genes (*MYB5*, anthocyanidin synthase (*ANS*), cellulase 1 (*CEL1*), and cellulase 2 (*CEL2*)). The study findings also revealed that changes in *FaGAPC2* and *FaGAPCp1* expression did not affect the ABA content in the overexpressed or RNAi fruit (Figure 4D and Figure 5D). FaABI1 is a negative ABA signaling regulator and involved in ABA-mediated fruit ripening in strawberries [33]. In our results, the expression of *FaABI1* was also affected by *GAPDH*, which may indicate that the effect of *GAPDH* on strawberry fruit ripening is related to the ABA signal. It has been reported that *Arabidopsis thaliana* mutants deficient in plastid glycolytic glyceraldehyde-3-phosphate dehydrogenase (*gapcp1gapcp2*) were ABA insensitive by altering the gene expression of the transcription factor *ABI4*, but the ABA levels were normal [35]. These findings suggested that GAPDH is an important negative factor involved in ABA and sucrose-mediated fruit ripening in strawberries with a new non-glycolytic function. ABA and sucrose promoted strawberry fruit ripening by inhibiting *FaGAPC2* and *FaGAPCp1* gene expression and this regulated the synthesis of sucrose and anthocyanin, transduction of ABA signaling pathway, and expression of ripening-related genes.

### 4.2. The Function of FaGAPC2 and FaGAPCp1 Is Closely Related to the Change in Oxidative Stress

GAPC is vulnerable to redox modification because its catalytic Cys occupies a large surface area in the N-terminal side of the α-helix and this increases the reaction rate and the thiol acidity [36]. Therefore, the modified GAPC possesses other new functions. This study focused on the relationship between the new function in fruit ripening in a strawberry and oxidative stress. To verify this hypothesis, different concentrations of H_2_O_2_ and DTT were used to treat strawberry fruit discs. The results showed that H_2_O_2_ treatment (1~8 mM) significantly inhibited the transcript level of the *FaGAPCp1* gene, while DTT treatment (4~8 mM) up-regulated the transcript levels of *FaGAPC2* and *FaGAPCp1* (Figure 6E). H_2_O_2_ is an important signal molecule in the ABA signal transduction pathway, and ABA can induce a large amount of H_2_O_2_ production in plants [37]. In this study, ABA and sucrose treatment was found to increase the endogenous H_2_O_2_ content in strawberry fruit (Figure 2C), as well as reduce the transcript level of *FaGAPC2* and *FaGAPCp1* genes (Figure 2A,B) and promote fruit ripening in strawberry. The changes in the transcript level of *FaGAPC2* and *FaGAPCp1* genes led to variations of fruit ripening progress in strawberries (Figure 4 and Figure 5). These results indicated that the transcript levels of *FaGAPC2* and *FaGAPCp1* were closely related with the oxidative stress, and the involvement of *FaGAPC2* and *FaGAPCp1* in ABA and sucrose-mediated strawberry fruit ripening affected the regulation of H_2_O_2_ directly or indirectly. Previous studies also indicate that various stresses can affect the function of GAPDH. Under oxidative stress, hydrogen peroxide promotes the interaction between GAPC and phospholipase phospholipase Dδ (PLDδ) to provide a molecular link between the stress signaling, energy metabolism, and growth control in *A. thaliana* [29]. In cadmium-treated *A. thaliana* roots, GAPC accumulated steadily in the nucleus, while NO content and cytosolic solute oxidation increased [6].

Apart from serving as an oxidative sensor, this study explored whether transient *FaGAPC2* and *FaGAPCp1* expression affected the endogenous H_2_O_2_ content and GSH/GSSG redox potential of strawberry fruit. The cellular balance of GSSG and GSH in plants provides a dynamic indicator of oxidative stress [38]. GSH is the main molecule that scavenges H_2_O_2_ and other reactive oxygen species in plants, and the increase of GSH content and the decrease of GSSG content indicate that plants may be subjected to oxidative stress [39]. Such a change leads to corresponding stress responses in plants. The results showed that overexpression of *FaGAPC2* and *FaGAPCp1* decreased the contents of H_2_O_2_ (Figure 6A) and the GSH/GSSG ratios, whereas silencing of *FaGAPC2* and *FaGAPCp1* increased the GSH/GSSG ratios (Figure 6D), suggesting that *FaGAPC2* and *FaGAPCp1* may regulate the redox state. Therefore, the function of *FaGAPC2* and *FaGAPCp1* and oxidative state influence each other, which affects fruit ripening in strawberry.

### 4.3. FaGAPCp1 Changes Ripening-Related Metabolite Synthesis

**Figure 8:**
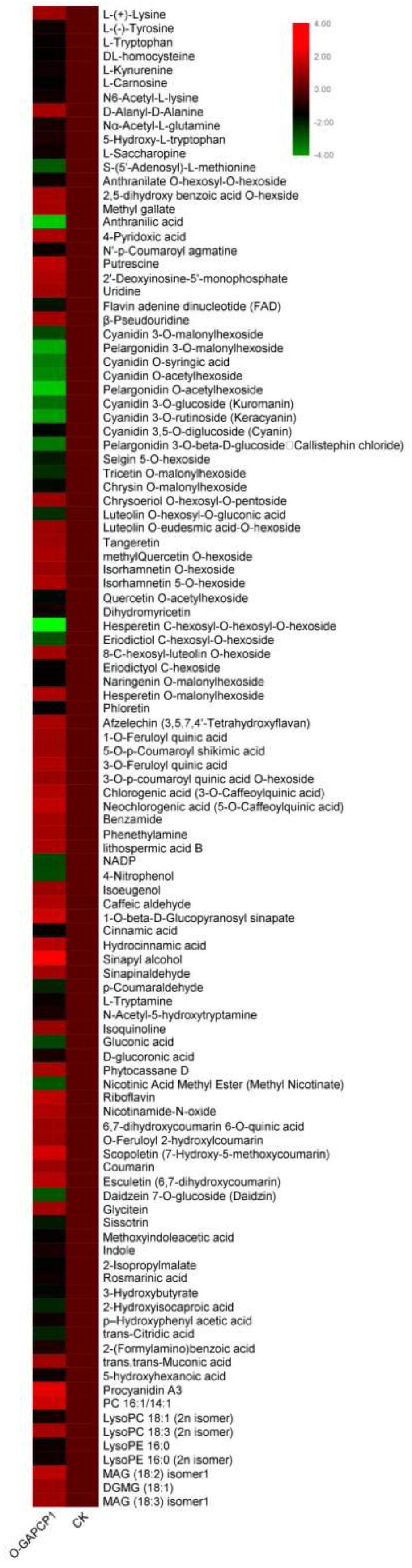
A heatmap of significantly differential metabolites in strawberry fruits after overexpression of *FaGAPCp1*. The value was determined by the relative content of metabolism, which corresponds to the log_2_ fold change (FC) of O-GAPCp1 compared to CK. The relative content variance of each compound was indicated by colors ranging from low (green) to high (red).

Previous studies on the multi-function of GAPDH mostly focused on GAPC, but very few have focused on GAPCp. GAPCp is defined as GAPDH located in plant plastids, however, it has been confirmed that GAPCp is not located in chloroplasts in angiosperms [2]. The main function of GAPCps in roots is to supply 3-phosphoglycerate (3-PGA) to the phosphorylated pathway of serine biosynthesis and stimulation of sugar biosynthesis in the aerial part of the plant [40]. In this study, the upregulation of *FaGAPCp1* was found to mainly affect the anthocyanins, flavonoids, organic acids, amino acids, and their derivative content in strawberry fruits (Figure 8).

Anthocyanins accumulated gradually during strawberry fruit ripening [41,42]. The main anthocyanins in strawberry fruits were cyanidin and pelargonidin [43]. Overexpression of *FaGAPCp1* inhibited the accumulation of both cyanidins and pelargonidin. Flavonoids are the main secondary metabolites in strawberry fruit, which are also involved in protection against various biotic and abiotic stresses. They also play a major role in the regulation of plant reproduction and act as signaling molecules [44]. It has been reported that plant genotype, growing condition, ripening stage, and harvesting are factors that influence the compositional variation of flavonoids in the fruit and the content of total flavonoids decreases during fruit ripening in strawberry [45]. In addition to anthocyanin glycosides, flavone, flavonol, and flavanone are the main flavonoids in the fruits [46]. In this study, transient overexpression of *FaGAPCp1* was found to increase flavonol relative content but decreased the flavones and flavanone relative content (Table S4). Amino acids are important precursors in plant biosynthesis. It not only affects the normal growth and development of plants, but also promotes the aroma and sugar content of fruits [47]. It has been reported that the amino acid content decreases gradually during the ripening process in strawberry reaching 48.39 at the young fruit stage and only 8.62 μg/g at the ripening stage [48]. These study results showed that the relative content of 11 amino acids and their derivatives decreased compared with the control (Figure 8). According to the report by Cascales–Minana [40], although the main function of GAPCps is in the serine biosynthesis pathway, there was no significant difference in serine content. *FaGAPCp1* changes the synthesis of ripening-related metabolites, especially anthocyanins, flavonoids, amino acids, and their derivatives, and this affects fruit ripening in strawberry.

In this study, ABA and sucrose treatment was found to induce a large amount of H_2_O_2_ production and inhibit the expression of *FaGAPC2* and *FaGAPCp1* in strawberry fruit. Transient knock-down of *FaGAPC2* and *FaGAPCp1* or overexpression accelerated or delayed fruit ripening in strawberries with a change in H_2_O_2_ level and GSH/GSSG redox potential. Oxidative stress regulates *FaGAPC2* and *FaGAPCp1*, and *FaGAPCp1* affects fruit ripening in strawberries through the metabolic changes of anthocyanins, flavonoids, organic acid, amino acids, and their derivatives. These results revealed that GAPC and GAPCp have a new non-glycolytic function which is a negative regulator involved in fruit ripening in strawberries. Meanwhile, *FaGAPC2*/*FaGAPCp1* and oxidative stress interplay closely with master coordinators of strawberry fruit ripening such as ABA and sucrose.

## Supplementary Materials

The following are available online at https://www.mdpi.com/2073-4425/11/5/580/s1, Figure S1: title, Table S1: title, Video S1: title.Table S1. Primers used for cloning in this study. Table S2. Primers used for plasmid construction in this study. Table S3. Primers used for real-time PCR in this study. Table S4. The the relative content of significantly differential metabolites in strawberry fruits after overexpression of FaGAPCp1.
