# Supplementary material for: Cytosolic/Plastid Glyceraldehyde-3-Phosphate Dehydrogenase Is a Negative Regulator of Strawberry Fruit Ripening
Source: Genes (Basel). 2020 May 21;11(5):580. doi: 10.3390/genes11050580 (PMC7291155; doi:10.3390/genes11050580)
Supplement: Supplementary file 1 [file genes-11-00580-s001.zip › supplemental table 1.docx]

Table S1. Primers used for cloning in this study

| Gene | Primer |
| --- | --- |
| FaGAPC2-F | CTCTCTTCGATCAGTCTCCATGGC |
| FaGAPC2-R | GCCTCGATCCAAGCTTAAGCC |
| FaGAPCp1- F | ATTGTGAGGTGCCGTTGT |
| FaGAPCp1- R | AGATCTGATCCTTTGCGTAC |
