# Supplementary material for: Cytosolic/Plastid Glyceraldehyde-3-Phosphate Dehydrogenase Is a Negative Regulator of Strawberry Fruit Ripening
Source: Genes (Basel). 2020 May 21;11(5):580. doi: 10.3390/genes11050580 (PMC7291155; doi:10.3390/genes11050580)
Supplement: Supplementary file 1 [file genes-11-00580-s001.zip › supplemental table 2.docx]

Table S2. Primers used for plasmid construction in this study

| Gene | Primer |
| --- | --- |
| pCAMBIA1301-FaGAPC2-F | CCGGAATTCGCATTCCTCATGGCCAAGATCAAG |
| pCAMBIA1301-FaGAPC2-R | GCTCTAGAGCAAGGAAGAAGTGGATGTTCAAGG |
| pTRV2-FaGAPC2-F | GCTCTAGAGAAGTGGAAGCACCACCATGAAC |
| pTRV2-FaGAPC2-R | CCGGAATTCACCGTGGTCATTAGACCCTCC |
| pCAMBIA1301-FaGAPp1-F | CCGGAATTCCCATGGCCAAGATCAAGATTGGC |
| pCAMBIA1301-FaGAPp1-R | GCTCTAGACCTCAGAGCCTCAATCAATTAACTGTGG |
| pTRV2-FaGAPp1-F | GCTCTAGAGCAGTGGAAGCATGGCGAG |
| pTRV2-FaGAPp1-R | CCGGAATTCGCCAGTGGAGCAAGACAGTG |
