# Supplementary material for: Cytosolic/Plastid Glyceraldehyde-3-Phosphate Dehydrogenase Is a Negative Regulator of Strawberry Fruit Ripening
Source: Genes (Basel). 2020 May 21;11(5):580. doi: 10.3390/genes11050580 (PMC7291155; doi:10.3390/genes11050580)
Supplement: Supplementary file 1 [file genes-11-00580-s001.zip › supplemental table 3.docx]

Table S3. Primers used for real-time PCR in this study

| Gene | Primer |
| --- | --- |
| MYB5- F | TATCAACCAGACGCAATA |
| MYB5- R | ATACACAAGCAACACTATG |
| PK- F | ATTGTGAGGTGCCGTTGT |
| PK- R | AGATCTGATCCTTTGCGTAC |
| ALDO- F | CGTGCTGTGCTCAAGATT |
| ALDO- R | AAGGACGTGGTGGTCATT |
| G6PDH- F | TCCTCCATCAGTATATCCATCT |
| G6PDH- R | TACGAGTCCATCCACCAT |
| NCED1- F | CGGAACCTGCTCGGTAGAAA |
| NCED1- R | AAGGAAGAAAGGCTCGCCAC |
| Actin- F | TGGGTTTGCTGGAGATGAT |
| Actin- R | CAGTTAGGAGAACTGGGTGC |
| CEL1- F | CCATACCCAAGTCCAATA |
| CEL1- R | ACGATAGCGAAGTTACAT |
| CEL2- F | TGATGCCTATGACAACTT |
| CEL2- R | ACGAGCCAATATACCAATA |
| ABI1- F | CAAGAGCCATTCTTTGTCGT |
| ABI1- R | TGGAATAATCCAGGGTTTCA |
| ANS- F | CGTGAGACCCAAAGAGGA |
| ANS- R | ATGCCGTGGTTGATAAGG |
| SS- F | GCCAATCATCTTCACAAT |
| SS- R | TTCTTACCATACCACTCAA |
| GAPC2-F | AAGAAGGTTGTCATCTCT |
| GAPC2-R | CTTGTATTCGTGCTCATT |
| GAPCp1-F | GGATACACCGATGAAGAT |
| GAPCp1-R | GTGGAACTAAGTGCTAATC |
